# Avian Pigment Pattern Formation: Developmental Control of Macro- (Across the Body) and Micro- (Within a Feather) Level of Pigment Patterns

**DOI:** 10.3389/fcell.2020.00620

**Published:** 2020-07-10

**Authors:** Masafumi Inaba, Cheng-Ming Chuong

**Affiliations:** Department of Pathology, Keck School of Medicine, University of Southern California, Los Angeles, CA, United States

**Keywords:** morphogenesis, melanocyte, regional specificity, integument, skin appendages, color, stripes, Evo-Devo

## Abstract

Animal color patterns are of interest to many fields, such as developmental biology, evolutionary biology, ethology, mathematical biology, bio-mimetics, etc. The skin provides easy access to experimentation and analysis enabling the developmental pigment patterning process to be analyzed at the cellular and molecular level. Studies in animals with distinct pigment patterns (such as zebrafish, horse, feline, etc.) have revealed some genetic information underlying color pattern formation. Yet, how the complex pigment patterns in diverse avian species are established remains an open question. Here we summarize recent progress. Avian plumage shows color patterns occurring at different spatial levels. The two main levels are macro- (across the body) and micro- (within a feather) pigment patterns. At the cellular level, colors are mainly produced by melanocytes generating eumelanin (black) and pheomelanin (yellow, orange). These melanin-based patterns are regulated by melanocyte migration, differentiation, cell death, and/or interaction with neighboring skin cells. In addition, non-melanin chemical pigments and structural colors add more colors to the available palette in different cell types or skin regions. We discuss classic and recent tissue transplantation experiments that explore the avian pigment patterning process and some potential molecular mechanisms. We find color patterns can be controlled autonomously by melanocytes but also non-autonomously by dermal cells. Complex plumage color patterns are generated by the combination of these multi-scale patterning mechanisms. These interactions can be further modulated by environmental factors such as sex hormones, which generate striking sexual dimorphic colors in avian integuments and can also be influenced by seasons and aging.

## Introduction

Many animals exhibit color patterns on their skin surface, which serve the function for inter- and intra-species communications. These patterns can be either highly vivid to draw attention from others in their local environment or drab to provide camouflage so they can blend into their environment. How these color patterns form is a fundamental question of scientific interest. Pigment patterns have been studied in different animals using different approaches. In zebrafish, relatively simple pigment patterns and strong genetics allowed scientists to discover genes related to pigment patterning and cell-cell interactions that govern patterning (Watanabe and Kondo, 2015; Irion et al., 2016; Patterson and Parichy, 2019). In mice, genetic studies have revealed many pigment-related mutants (Bennett and Lamoreux, 2003). Recent progress of genetic and genome-wide analysis led to the identification of several important genes involved in pigment patterning in non-model animals, such as the cheetah, horse, and chipmunks (Kaelin et al., 2012; Imsland et al., 2016; Mallarino et al., 2016).

**FIGURE 1:**
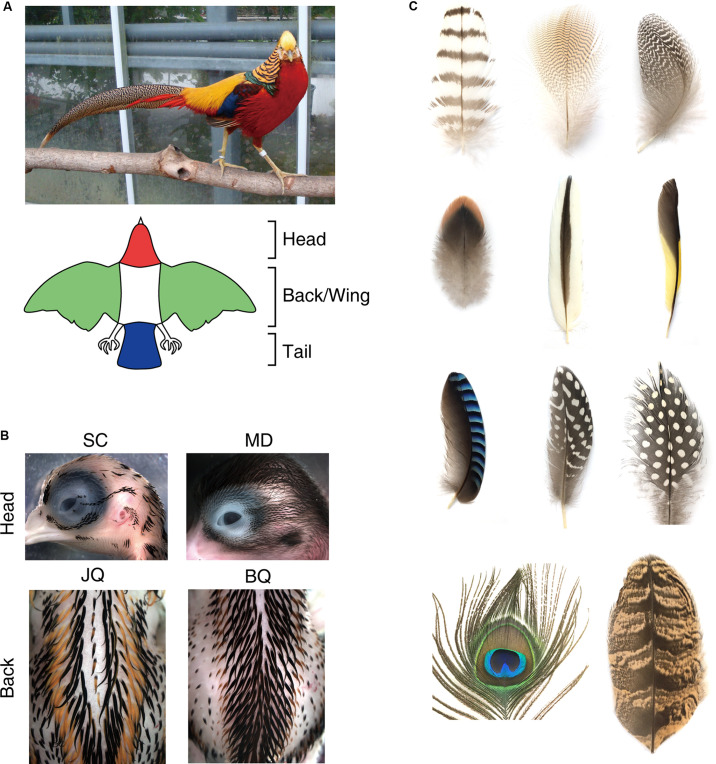
Macro- (across the body or within a skin domain) and micro (within a feather) color patterns in the bird. **(A)** Color patterns across the whole body. Upper: a male golden pheasant. Lower: a schematic drawing showing a typical whole color pattern in birds. **(B)** Pigment patterns within domains. Upper: pigment patterns on the head in an E12 Sicilian Buttercup chicken (SC) and E16 Mallard duck (MD). Lower: distinct pigment stripe patterns in E10 Japanese quail (JQ) back skin and E13 Bobwhite quail (BQ). **(C)** Diversity of pigment patterns within a feather. Photos by M. Inaba.

Although the complex pigment patterns and accessibility for analyses make birds a fascinating model to study morphogenesis, little is known about patterning mechanisms. Here we summarize recent results from our and other’s studies. Bird’s colorations emerge in their skin and/or feathers as striped, spotted, and more complex patterns, which provide us with an excellent platform to study biological pattern formation. Birds have a variety of ways of producing colors (several types of pigments and structural color) as well as a multiscale hierarchy in their macro- (across body/domains) to micro- (within feathers) pattern distributions. The largest macro-pigment pattern covers the whole-body pattern (Figure 1A). Most birds exhibit a specific color on a specific region, such as the head, back, wing, and tail. This implies that a long-range patterning mechanism enables the specification of each domain across the whole body. Each domain often exhibits regular pigment patterns, such as stripes with different sizes (Figure 1B). Lastly, the pigment patterns within a feather form micro-patterns (Figure 1C). So far, many attempts to understand the mechanism of avian pigment patterning have been performed, especially in different domains and within feather patterns, from the viewpoint of genetics, developmental biology, or mathematical biology. Domestic fowl genetics has brought understanding into the molecular basis of pigmentation (Kerje et al., 2003; Ishishita et al., 2018; Andersson et al., 2020). Developmental biology approaches using transplantation of embryonic tissues have revealed the important interactions between pigment cells and skin tissues that contribute to pigment patterns (Haupaix et al., 2018; Inaba et al., 2019b). In this review, we discuss the known mechanisms of avian pigmentation and how the pigmentation is regulated to make spatial patterns at different scales (skin domain and feather level). We also present possible future directions that can help unravel the complex avian pigment patterns.

## Molecular Mechanism of Colorations: Melanin, Non-Melanin Chemical Pigment, and Structural Colors

Bird colorations are derived from pigments and structural colors. The pigments are chemical substances that directly absorb and reflect specific wavelengths of light. Birds exploit different groups of pigments: melanins, produced by melanocytes, are the main determinant of the skin and feather color (Galván and Solano, 2016). There are two melanin types: eumelanin (black) and pheomelanin (yellow to orange). Switching melanin type and the regulation of melanin density play an important role in forming pigment patterns in a wide range of animals (Hoekstra, 2006). There are also chemical pigments that are not derived from melanocytes. The non-melanin pigments include carotenoids (yellow, orange, red, and purple), pterins (yellow and red), porphyrins (red, brown, and green), or psittacofulvins (yellow and red) (Lucas and Stettenheim, 1972; Galván and Solano, 2016; Lopes et al., 2016; Cooke et al., 2017).

In addition, structural colors are also important factors that confer shiny colors on birds. Generally, structural colors occur by the interaction of light with nanoscale structures, such as melanosomes or keratin filaments in feathers (Yoshioka and Kinoshita, 2002; Zi et al., 2003; Maia et al., 2012). A combination of chemical and structural colors can expand the variation of colors displayed as seen in parrots (Cooke et al., 2017). In this minireview, we will focus more on the control of melanin pigments.

In vertebrates, melanins are produced by melanocytes (Schartl et al., 2016). Melanocytes in the skin are derived from neural crest cells (NCCs), while those in the retina (retinal pigment epithelium) are derived from optic neuroepithelium. Melanocyte progenitor cells (melanoblasts) express microphthalmia-associated transcription factor (MITF), a master regulator of melanocyte development. During maturation, melanocytes express tyrosinase (TYR) and tyrosinase-related protein 1 (TYPR1), enzymes that produce melanin pigment. Melanocytes contain organelles called melanosomes where melanins are synthesized and accumulated. Mature melanosomes are transferred to adjacent keratinocytes through melanocyte dendrites. So far, different models of melanosome transfer have been proposed (Wu and Hammer, 2014). A recent study using live imaging of chicken embryonic skin revealed that melanosomes are transferred by vesicles derived from the melanocyte plasma membrane (Tadokoro et al., 2016).

The expression of melanins can be modulated by many factors. For hormonal regulation of melanogenesis, the melanocortin pathway has been well studied (Wolf Horrell et al., 2016). Melanocortins, including adrenocorticotropic hormone (ACTH) and several types of melanocyte-stimulating hormone (MSH), are produced in the pituitary gland and bind to the melanocortin 1 receptor (MC1R), which is a G protein-coupled receptor expressed by melanocytes. Binding of αMSH to the MC1R increases cellular cyclic adenosine monophosphate (cAMP), leading to eumelanin synthesis. The agouti signaling protein (ASIP) is a secreted peptide that inhibits MC1R signaling by antagonizing αMSH binding. Binding of ASIP to MC1R leads to preferential pheomelanin synthesis. Pheomelanin production also requires cysteine uptake into melanocytes via the xCT transporter (Chintala et al., 2005). Cysteine availability may be affected by environmental oxidative stress (Galván et al., 2017), leading to color changes. ASIP is preferentially expressed in the ventral skin, and its level and spatial domain can affect the survival fitness of wild animals (Millar et al., 1995; Manceau et al., 2011; Ceinos et al., 2015).

## Macro-Patterns: Color Patterns Across the Whole Body and Within a Skin Domain

Bird pigment patterns across the whole body include stripes, spots, patches, or more complex patterns. This is achieved at the whole-body level (Figure 1A), either by pigment patterns in a skin domain (Figure 1B), within a feather (Figure 1C), or a combination of these two locations. Here, we focus on the pigment pattern arising in the skin domain. Interestingly, striped patterns are the most prominent pigment patterns observed in the bird’s skin domain. Stripes often emerge in embryonic skin domains, as seen in the head and back skin (Figure 1B). The patterns of stripes vary among related species, implying that they share a common pattern control mechanism with minor genetic changes that function in a modulatory capacity.

**FIGURE 2:**
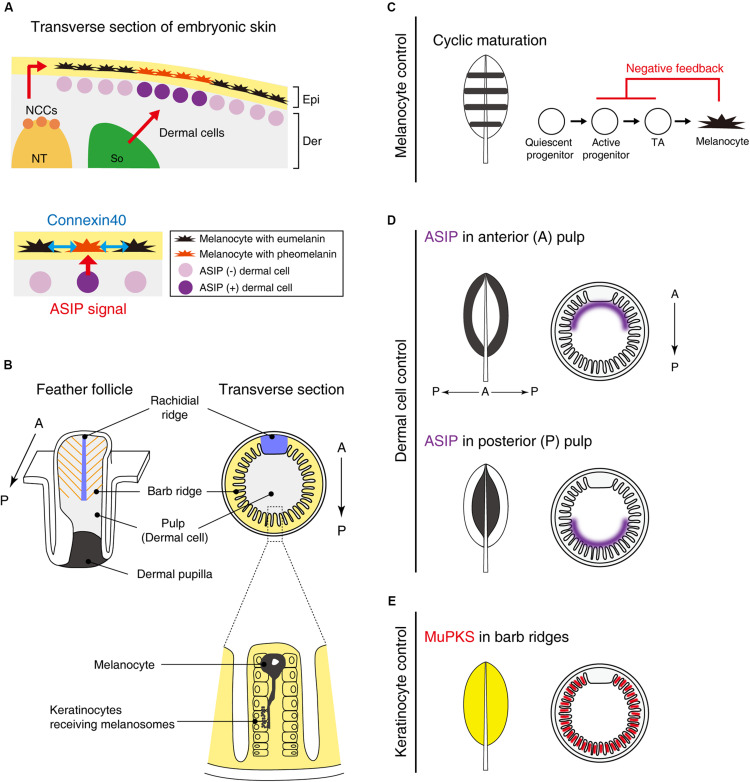
Putative molecular mechanisms in macro- and micro-pigment pattern formation. **(A)** The embryonic development of Japanese quail skin (Left). Neural crest cells (NCCs) delaminate from the dorsal part of the neural tube (NT). Some NCCs differentiate into melanoblasts and emigrate to their destination in the epidermis (Epi). A part of the somite (So) differentiates to dermis (Der). Dermal cells in the vicinity of melanocytes secrete ASIP to switch the melanin synthesis pathway from eumelanin to pheomelanin. Dermal cell–melanocyte interactions confer striped pigment patterns (Lower) (Haupaix et al., 2018). In addition, melanocytes interact with each other via connexin40 (blue arrows) to refine the size of the pigmentation stripes (Inaba et al., 2019b). **(B)** Schematic drawing of a feather follicle. Melanocytes are located within barb ridges and transport its melanosome into adjacent keratinocytes (Lin et al., 2013). **(C–E)** Schematic drawing showing that interactions between melanocytes and their environments (dermis/epidermis) form a variety of feather pigment patterns. **(C)** Melanocyte intrinsic banded pigment pattern. Cyclic maturation of the progenitor cells gives rise to banded pigment patterns on the feather vane. To accomplish this oscillation, negative feedback from mature melanocytes to active progenitors and transit amplifying cells (TA) is assumed. **(D)** Dermal cell instructive pigment pattern. ASIP is expressed in either side of the anterior or posterior pulp comprised of dermal cells, leading edge, or central pigmented patterns (Lin et al., 2013). **(E)** Feather keratinocytes can confer the pigmentation. MuPKS, polyketide synthase, is expressed in barb ridges and deposits yellow psittacofulvin pigments (Cooke et al., 2017).

Galliform birds such as guineafowl, pheasant, chicken, and quail often show stripe patterns on their back skin at embryonic and juvenile stages. During development, melanoblasts migrate along the dorsoventral pathway between the epidermis and dermis and eventually incorporate into the epidermal layer (Figure 2A). Prior to melanin synthesis, melanoblasts are committed to producing eumelanin, which is marked by expression of glutathione s-transferase (Nataf et al., 1993, 1995; Niwa et al., 2002). While the mechanism of this specification has not been discerned yet, this process can be regulated by melanocytes autonomously or modulated by surrounding tissues. Therefore, the molecular mechanism of pigment patterning must clarify the role of each tissue in this process.

Tissue interactions can be studied utilizing tissue grafting techniques between different avian species. In classical experiments using chicken-quail chimera, when Japanese quail neural crest was transplanted into White Leghorn chickens which show no pigmentation, a quail-like striped pigment pattern appeared in the host chicks (Kinutani and Le Douarin, 1985). This result suggests that melanocytes likely have information that guides pigment patterning. In another study, Japanese quail wing buds prior to melanocyte immigration were transplanted into guineafowl embryos (Richardson et al., 1991). This experiment placed guineafowl melanocytes surrounded by quail tissues (epidermis and dermis). The expected results predicted two possible outcomes: (1) if the host shows a guineafowl-like pigment pattern, it would suggest that melanocytes of different genotypes can respond differently to environmental cues shared among different species. (2) If the host shows a quail-like pigment pattern, it would suggest that a prepattern guiding pigment patterning is present in environmental tissues (epidermis or dermis). The result of this transplantation produced the latter result. Such hetero-species tissue recombination analysis should be valuable to identify the molecular cues needed for pigment patterning.

Recently, somite transplantation experiments among galliform birds showed that longitudinal stripe patterns developing on embryonic back skin correlated with dermal ASIP expression patterns. Hence, pigment stripe patterning was determined by the somite species of origin (Haupaix et al., 2018; Haupaix and Manceau, 2020) (Figure 2A). Loss-of-function mutant quails with a shortened ASIP expression period display a thinner yellow stripe width, whereas the gain-of-function mutant with a prolonged-expression period expands the yellow stripe width. Furthermore, by grafting Japanese quail somites into pheasant embryos, the quail specific ASIP expression pattern was found to be induced in the host. These results led the authors to suggest that the stripe pattern is determined by the regulation of ASIP in dermal cells derived from somites. Yet how these dermal cells generate the striped agouti expression pattern was not addressed yet.

Although somites can play an important role in bird stripe patterning, other tissues may contribute to the patterning in different ways. Quail melanocytes purified in *in vitro* culture were transplanted into White Leghorn chickens. Interestingly, host chickens initially express ASIP in longitudinal stripes but ASIP disappears soon after E6 (Inaba et al., 2019b). In the chicken host, quail melanocytes form periodic pigment patterns without directionality along the longitudinal axis. This suggests that melanocytes still have an ability to form periodic patterns autonomously but the stripe directionality is largely dependent on the striped ASIP expression pattern. The stripe width is regulated not only by levels of ASIP but also by melanocyte interactions in a connexin-dependent manner. Connexins can comprise gap junctions that directly connect neighboring cells, enabling cell–cell communications via small molecules, such as ions and second messengers. Connexin40 is expressed in both eumelanin and pheomelanin producing melanocytes. Because the melanocytes contact each other in the skin with filopodia, connexin40 is expected to comprise gap junctional channels between melanocytes (Figure 2A). Over-expression of connexin40 in quail melanocytes leads to the expansion of yellow regions with a corresponding reduction of black regions. In contrast, inhibition of connexin40 by expressing a dominant negative form reduced the yellow and expanded the black regions.

It should be noted that the role of gap junctions in stripe patterning has been studied in zebrafish before. Zebrafish stripes are formed by pigment cell interactions that satisfy the Turing-type patterning (reaction-diffusion system) requirement (Nakamasu et al., 2009; Inaba et al., 2012; Hamada et al., 2014). A recent transgenic zebrafish study showed that unidirectional current flow between melanocytes mediated by gap junctions is sufficient to produce stripe patterning (Usui et al., 2019). In the future, it is important to understand how gap junction communication is involved in generating the self-organizing pigment patterning process.

Taken together, ASIP expression may present a prepattern that guides the global orientation of stripes, while self-organizing melanocytes involve the local patterning of stripes. Such global and local patterning mechanisms are often exploited combinatorially to achieve robust and plastic development, e.g., feather bud arrangement (Bailleul et al., 2019; Ho et al., 2019; Inaba et al., 2019a). In studying the patterning process, one has to bear in mind that both autonomous and non-autonomous mechanisms may be used together or in different contexts, and that a pattern (e.g., stripes) can be generated by multiple mechanisms.

## Micro-Patterns: Color Patterns Within a Feather

Pigment patterning within a feather vane is fascinating, as spots, stripes, chevrons, rings, or combinations of these patterns can be formed (Figure 1C). Although feathers are cyclically replaced to maintain their functions throughout a lifetime, they can retain identical pigment patterns in each feather generation. On the other hand, feather pigment patterns can be modulated in response to intrinsic and extrinsic factors. The feather pigment pattern varies depending on the characteristics of the region where feathers can grow (tract, pterylae). This suggests that the patterning mechanism is influenced by positional information that determines body regional specificity. Further, sexual maturation can alter feather morphology, texture, or pigment patterns to attract a mate. The sex hormones indeed determine the feather phenotypes; however, the molecular mechanisms that convert the hormonal signal to feather coloring remain to be elucidated (Widelitz et al., 2019).

To appreciate patterning within a feather, we need to appreciate the topology of the platform (Lucas and Stettenheim, 1972; Yu et al., 2004). The follicle structure (Figure 2B) is formed by the invagination of feather primordia toward the dermal layer. The feather follicle epithelial sheet periodically invaginates along the circumference of the follicle, forming future feather branches called barb ridges. The anterior part of the epithelial sheet is specialized within the rachidial ridge for future feather shaft development. The follicles contain dermal cells: those in the distal follicle become pulp and those in the proximal follicle become the dermal papilla, which plays a critical role in feather regeneration. In the resting phase of feather development, melanoblasts are localized in the feather follicle papilla ectoderm. During the growth phase, melanoblasts are located at the lower bulge zone, close to the feather follicle base. Melanoblasts migrate upward into barb ridges and then differentiate into mature pigmented melanocytes (Lin et al., 2013). Mature melanocytes located in barb ridges transport their melanosomes to keratinocytes to form the micro-pigment patterns (Lucas and Stettenheim, 1972).

At least two major mechanisms to achieve unpigmented (white color) under the control of melanocyte activity have been investigated (Lin et al., 2013). The first is the presence/absence of melanoblasts. White Leghorn chickens completely lose the progenitor cells at a late stage of embryogenesis (Jimbow et al., 1974), resulting in entirely white feathers. When melanoblasts are controlled spatiotemporally, more complex pigment patterns emerge. Barred Plymouth Rock chickens, which have mutations in the tumor suppressor gene, CDKN2A (Hellström et al., 2010), display banded pigment patterns. The cis-regulatory mutations upregulate the expression of CDKN2A, which leads to melanoblasts reduction, not apoptosis. The melanocyte maturation zone cyclically travels from the posterior to the anterior side during feather growth from the distal to the proximal end. This cyclic presence/absence of melanoblasts driven by a negative feedback system results in the black/white banded patterns oriented along the proximal-distal axis (Figure 2C).

The second mechanism is the control of melanocyte melanin synthesis by adjacent dermal cells (Figure 2D). The pulp derived from dermal cells heterogeneously expresses ASIP. When ASIP expression is localized in the anterior side of feather pulp, the central feather exhibits a white color while the feather edge displays a black color. How this polarized ASIP expression in pulp fibroblasts is regulated is unknown, but we know it is a sexually dimorphic process (Lin et al., 2013). Administration of estrogen to male chickens can induce a female-specific ASIP expression pattern that causes female-like pigmentation (Oribe et al., 2012). Through sex-hormone regulation, birds can reset feather colors and forms by molting and regenerating new feathers at different life stages (Widelitz et al., 2019).

In addition to the roles played by melanocytes and dermal cells, the keratinocytes also play a role in pigmentation (Figure 2E). One example is polyketide synthase (MuPKS), an enzyme that produces yellow polyene pigments called psittacofulvins. MuPKS is expressed in keratinocytes of barb ridges during follicle formation, which confers yellow pigmentation on budgerigar feathers (Cooke et al., 2017).

## Future Directions

Avian color patterns provide a wonderful patterning model that acts at different spatial scales (Figure 1). In this minireview, we covered some classical studies, recent experiments examining tissue interactions, and lines of experimentation providing some molecular insights into pigment patterning (Figure 2). With the advance of current technologies, we expect a new synthesis will help reveal the organizing principles of complex patterns. We think further advances will need to include the following approaches.

(1) Cell and developmental biology approaches: More analyses of melanocyte stem cell status, proliferation, migration and differentiation, and their interactions with adjacent tissues within the embryo and regenerating feather follicles (Lin et al., 2013; Haupaix and Manceau, 2020) will help discern cellular mechanisms that underlie pigment patterning. This will also be aided by higher resolution time-lapse imaging analyses of cell interactive behaviors.
(2) Systems biology approaches: Recent advances in the genome-wide analysis would enable us to detect more novel genes related to pigment patterns both from domestic and wild birds. Bulk and single-cell transcriptomics, ATAC, Chip-seq provide a new possibility to understand epigenetic changes and fate specification which can be further evaluated with promoter-reporter analyses for melanogenesis pathway members and factors that activate them.
(3) Genetic approaches: Some chicken variants show unique color patterns and can provide clues to the patterning process (Cooke et al., 2017; Andersson et al., 2020).
(4) Develop a biological database patterning model: Previously, mathematical modeling based on a reaction-diffusion system has provided diverse pigment patterns that simulate the real feather pigment patterns (Prum and Williamson, 2002). While mathematical models help to highlight possible patterning principles, it is important to examine the actual biological processes that generate pigment patterns and synthesize a model based on experimental results. With these investigations, we hope to advance our understanding of the development, regeneration, and evolution of feather forms and pigment patterns (Chen et al., 2015).

## Author Contributions

MI and C-MC wrote the review. MI made figures. Both authors contributed to the article and approved the submitted version.

## Conflict of Interest

The authors declare that the research was conducted in the absence of any commercial or financial relationships that could be construed as a potential conflict of interest.
